# The Landscape of ALK-Rearranged Non-Small Cell Lung Cancer: A Comprehensive Review of Clinicopathologic, Genomic Characteristics, and Therapeutic Perspectives

**DOI:** 10.3390/cancers14194765

**Published:** 2022-09-29

**Authors:** Valeria Cognigni, Federica Pecci, Alessio Lupi, Giada Pinterpe, Chiara De Filippis, Cristiano Felicetti, Luca Cantini, Rossana Berardi

**Affiliations:** Clinica Oncologica, Università Politecnica delle Marche, AOU Ospedali Riuniti, 60126 Ancona, Italy

**Keywords:** non-small cell lung cancer (NSCLC), anaplastic lymphoma kinase (ALK), ALK inhibitors, resistance mechanism, liquid biopsy

## Abstract

**Simple Summary:**

In recent years, prognosis of non-small cell lung cancer (NSCLC) patients significantly improved thanks to the introduction of tyrosine kinase inhibitors (TKIs) in clinical practice. ALK-rearranged NSCLC patients benefit from treatment with ALK inhibitors (ALK-i), which have shown a greater efficacy and a better intracranial activity than chemotherapy. Comparative studies between next-generation ALK-i are still lacking and clinicians are looking for reliable tools to determine which drug suits best for each patient. The aim of this review is to deepen the role of clinical and pathological characteristics influencing patients’ prognosis during treatment with ALK-i and to provide an overview of molecular mechanisms of ALK-i resistance. In this setting, liquid biopsy may play an important role in predicting tumor response and monitoring resistance mutations. We will summarize ongoing trials developing new ALK-i or combinations between ALK-i and other agents, which may represent future scenarios in the field of NSCLC research.

**Abstract:**

During the last decade, the identification of oncogenic driver mutations and the introduction of tyrosine kinase inhibitors (TKIs) in daily clinical practice have substantially revamped the therapeutic approach of oncogene-addicted, non-small cell lung cancer (NSCLC). Rearrangements in the anaplastic lymphoma kinase (ALK) gene are detected in around 3–5% of all NSCLC patients. Following the promising results of Crizotinib, a first-generation ALK inhibitor (ALK-i), other second-generation and more recently third-generation TKIs have been developed and are currently a landmark in NSCLC treatment, leading to a significant improvement in patients prognosis. As clinical trials have already demonstrated high efficacy of each ALK-i, both in terms of systemic and intracranial disease control, comparative studies between second and third generation ALK-i are still lacking, and primary or secondary ALK-i resistance inevitably limit their efficacy. Resistance to ALK-i can be due to ALK-dependent or ALK-independent mechanisms, including the activation of bypass signaling pathways and histological transformation: these findings may play an important role in the future to select patients’ subsequent therapy. This review aims to provide an overview of underlying molecular alterations of ALK-i resistance and point out promising role of liquid biopsy in predicting tumor response and monitoring resistance mutations. The purpose of this review is also to summarize current approval for ALK-rearranged NSCLC patients, to help clinicians in making decisions on therapeutic sequence, and to deepen the role of clinicopathological and genomic characteristics influencing patients’ prognosis during treatment with ALK-i.

## 1. Introduction

Lung cancer is the second most commonly diagnosed tumor worldwide, considering both sexes, and representing the leading cause of cancer-related death with 1.8 million deaths in 2020. Despite recent therapeutic advances due to the introduction of target therapy and immunotherapy, 5-year survival remains poor (10–20%) [1].

Non-small cell lung cancer (NSCLC) represents about 85% of all lung cancer diagnosis and, in the last ten years, an increasing number of activating oncogene mutations have been discovered in this setting: the most frequent ones are *KRAS* (20–30%), *EGFR* (10–15% of Caucasian patients and up to 40% of Asian patients) and *ALK* (anaplastic lymphoma kinase) gene. *ALK* gene rearrangement is found in 3–5% of all NSCLC cases and is mostly represented by an ALK translocation, which usually involve the gene coding for the Echinoderm Microtubule-associated protein Like 4 (*EML4*) [2]. This molecular rearrangement results in the production of a fusion protein with a constitutive kinase activity that continuously stimulates cell proliferation and survival and inhibits apoptosis by modulating intracellular signaling pathways. 

ALK rearrangement is generally correlated with adenocarcinoma histology and patients with this gene mutation are mostly younger, with light- or never-smoking history. Patients with ALK rearrangement present a high risk for brain metastasis, which occur in approximately 30% of cases at the time of diagnosis [3,4,5]. 

During the last decade, clinical outcomes of ALK-rearranged advanced NSCLC patients significantly improved due to development of small molecules that specifically inhibits ALK (ALK inhibitors, ALK-i). Crizotinib was the first one to be approved in 2011 by FDA as upfront treatment [6], then many tyrosine kinase inhibitors (TKIs) of second and third generation have been developed in clinical trials and have replaced crizotinib due to their greater disease control and intracranial activity. However, patients inevitably experience disease progression because of the occurrence of acquired resistance to ALK-i mediated by multiple molecular mechanisms [7], such as secondary ALK mutations or activation of other molecular pathways. The availability of these drugs revealed the need for appropriate molecular diagnostics to identify specific resistance mechanisms and to guide the choice of subsequent treatment for ALK-rearranged NSCLC patients. 

In this review, we discuss the biology of *ALK*-gene alterations and the mechanisms of resistance to ALK-i in NSCLC patients. Furthermore, we aim to provide an overview of current clinical trials exploring combination therapeutic strategies to overcome acquired resistance to ALK-i. Moreover, we will focus on the applications of liquid biopsy in clinical practice as a key weapon for the detection of mechanisms of resistance. 

## 2. Oncogenic ALK Mutations: Insight into Molecular Alterations 

ALK is a transmembrane receptor tyrosine kinase (RTK) encoded by the *ALK* gene localized on chromosome 2, belonging to the superfamily of insulin receptor kinase. The RTK comprises a unique extracellular ligand-binding domain, a transmembrane domain and an intracellular tyrosine kinase domain. The extracellular portion of ALK contains two MAM domains (named after meprin, A-5 protein and receptor protein tyrosine phosphatase mu), a glycine-rich region (GR) and a LDLa domain. The function of ALK is not fully understood, but it probably contributes to the development of the nervous system. In human adults, ALK expression is mainly localized to the central nervous system (CNS), testes and small intestine [8] and the identification of ALK ligand(s) remains a critical issue [9]. Three of the suspected ALK ligands are pleiothropin [10], midkine [11] and augmentor [12], which showed in vitro binding specificity to ALK and a temporal and spatial expression pattern similar to that of ALK.

ALK regulates several pathways involved in cellular survival, replication and apoptosis, including the PI3K/AKT/mTOR, MAPK and STAT3 pathways, and it contributes to initiation and progression of different human tumours and cell lines, such as lymphomas, neuroblastoma and NSCLC [13,14].

Three types of mutations that lead to alteration of intracellular signaling pathways, have been described in the *ALK* gene: rearrangement (ALK-R), amplification (ALK-A) and point mutation. In NSCLC, the most common alteration detected is a translocation with another partner gene leading to a fusion between the kinase domain of ALK and the amino-terminal portion of different protein partners, promoting the activation of downstream signaling pathways, and resulting in increased proliferation and cell survival. In 1994, ALK-R was firstly identified in anaplastic large-cell lymphoma, as a nucleophosmin fusion partner (NPM-ALK) resulting from a (2;5) chromosomal translocation [13]. Subsequently, ALK-R has been described in other solid cancers, leading to an abnormal ALK overexpression. The oncogenic mechanism of ALK-A was first described in 2002 in neuroblastoma cell lines and has been discovered also in NSCLC, while point mutations of the ALK locus, most commonly in the kinase domain, have been reported mostly in patients with neuroblastoma and thyroid cancer [15,16]. In the NSCLC setting, point mutations detain a main role in conferring resistance to ALK-i, as described below.

In the context of advanced NSCLC, ALK-R are mutually exclusive with EGFR or KRAS activating mutations, and generally involve a common breakpoint in exon 20 of *ALK* locus. *EML-ALK* fusion gene was firstly identified in 2007 and represents the most frequent rearrangement in NSCLC patients (accounting for approximately 95% of ALK fusion variants): it results from a translocation, specifically involving an inversion on the short arm of chromosome 2 that connects the 5′ end of the *EML4* gene with the 3′ end of the *ALK* gene. 

EML4 belongs to the family of echinoderm microtubule-associated protein (EMAP)-like proteins and is composed of an N-terminal basic region, a hydrophobic EMAP-like protein domain and a tryptophan-aspartic acid (WD) repeat region. EML4 detains an important function for the formation of an intact microtubule network and plays an essential role in cell survival and proliferation [17,18].

**Figure 1:**
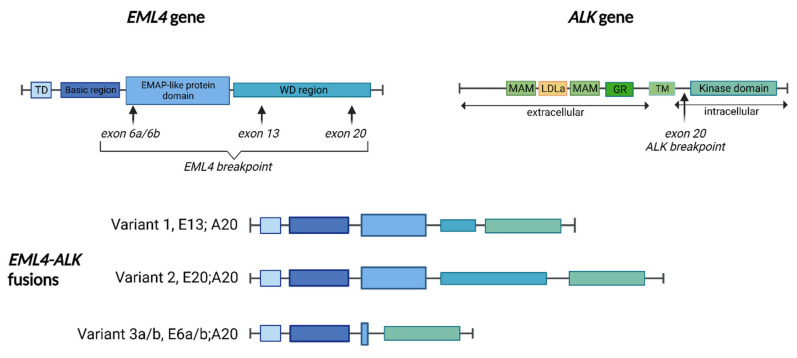
The *EML4-ALK* gene fusions: three major EML4-ALK variants are represented here, showing where the ALK kinase domain is inserted into the EML4 protein. Breakpoints in *EML4* and *ALK* that generate different variants are marked with black arrows. Abbreviations: ALK, anaplastic lymphoma kinase; EMAP, echinoderm microtubule-associated protein; EML4, echinoderm microtubule-associated protein like 4; GR, glycine-rich region; LDLa, low-density lipoprotein alpha domain; MAM, meprin, A-5 protein, and receptor protein-tyrosine phosphatase mu; TD, trimerisation domain; WD, tryptophan-aspartic acid region; TM, trans-membrane region.

The EML4-ALK fusion protein detains a potent oncogenic activity, both in vitro and in vivo, by activating an intrinsic tyrosine kinase and by constitutively triggering MAPK, JAK-STAT and PI3K/AKT pathways. Several variants of the EML4-ALK mutation have been described, based on different breakpoint on EML4, and they are localized in cytoplasm or to microtubules (Figure 1). The most common is variant 1 (33%), in which exon 13 of EML4 is fused with exon 20 of ALK (E13; A20), while variant 2 (10%) and variant 3a/b (29%) involve exon 20 and exon 6a or 6b of EML4, respectively, fused with exon 20 of ALK. The identification of specific EML4-ALK variants may have prognostic and predictive roles. For example, a Japanese study, which retrospectively evaluated the ALK variants of 35 patients treated with crizotinib, distinguished patients with variant 1 (54%) and nonvariant 1, demonstrating the better efficacy of crizotinib in patients with ALK variant 1 compared with the others [19]. In addition, other studies have shown that EML4-ALK variant 3 is significantly associated with a better response to lorlatinib, but also with the development of resistance mutations to ALK, particularly to G1202R [20,21].

Although EML4-ALK with different fusion breakpoints in *EML4* gene remains the major fusion variant in ALK-rearranged NSCLC, at least other 90 distinct fusion partners have been identified with NGS method in ALK-rearranged NSCLC [21]. Among these, the most important partner genes other than EML4 are *KIF5B, KLC1, TFG, TPR, HIP1, STRN, DCTN1, SQSTM1, NPM1, BCL11A* and *BIRC6*. Targeted therapeutic agents have demonstrated clinical efficacy in treating NSCLC patients with EML4-ALK gene fusion, but also in treating tumors with ALK fused with other partner genes, including NPM1 and BCL11A [22].

## 3. ALK-TKIs: Current Approvals and Daily Clinical Practice

**Figure 2:**
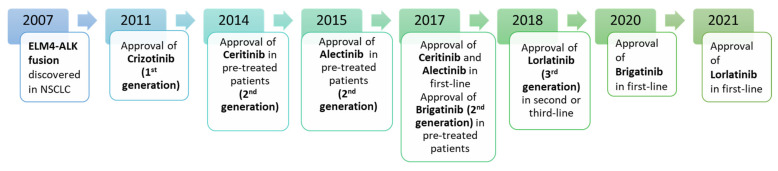
Approval timeline of currently available ALK inhibitors.

Currently, several ALK-i have been approved as standard treatment for ALK-rearranged NSCLC, including crizotinib (first generation), alectinib, ceritinib, brigatinib, ensartinib (second generation), and lorlatinib (third generation) (Figure 2). These agents have significantly improved patients’ survival outcomes, by inducing long-term responses, and new agents, comprising four-generation drugs, are being studied in ongoing clinical trials.

The anti-tumor mechanism of ALK-i is related to their activity as ATP analogue inhibitors of ALK, because of their binding to the ATP-binding pocket of the intracellular tyrosine kinase domain. Despite slight differences of each ALK-i, autophosphorylation of ALK is inhibited and downstream signal transduction pathways described above are blocked [16].

### 3.1. Crizotinib

Crizotinib, a first-generation inhibitor, was the first ALK-i approved for the treatment of ALK-rearranged NSCLC in 2011. It demonstrated its efficacy in first-line setting in the phase III study, PROFILE 1014, in which it was compared to pemetrexed plus platinum doublet chemotherapy: the median PFS was 10.9 months in the crizotinib arm and 7.0 months in the chemotherapy arm (HR: 0.45, 95% CI 0.35–0.60, *p* < 0.001), with a significant activity also in ORR (74% vs. 45%, respectively, *p* < 0.001) [23]. This benefit has not been confirmed in OS, probably because of cross-over and use of next-generation TKIs in subsequent lines. Moreover, the difficulty of blood-brain barrier penetration of crizotinib makes the brain a common site of disease progression resulting in a resistance to this drug mostly manifested within a year [24].

### 3.2. Ceritinib

The need to improve the activity for CNS disease and overcome both intrinsic and acquired mechanisms of resistance led to the development of second generation’s ALK-i. Ceritinib demonstrated good intracranial activity and a significant PFS advantage against first-line chemotherapy (16.6 months vs. 8.1 months, HR: 0.55, 95% CI 0.42–0.73, *p* < 0.00001), as showed in the ASCEND-4 study [25].

### 3.3. Alectinib

Alectinib was approved by the FDA in December 2015 for patients who had progressed on crizotinib, with full approval in 2017 as a first-line treatment for ALK-positive NSCLC. Similar to ceritinib, alectinib provided higher response rates and excellent brain penetrance [26]: in the phase 3 ALEX trial, it demonstrated a significant benefit in PFS (median PFS 34.8 vs. 10.9 months, HR: 0.43, 95% CI 0.32–0.58) and also in OS (5-year OS rate of 62.5% vs. 45.5%) when compared to crizotinib in first-line, becoming one of the most commonly used first-line therapies [27].

### 3.4. Brigatinib

Similarly, in the phase III ALTA-1L study, brigatinib, a potent second generation ALK-i, demonstrated superior activity compared to crizotinib in first line setting in survival and efficacy terms (3-year PFS for brigatinib of 43% vs. 19% for crizotinib, HR: 0.48, 95% CI 0.35–0.66, *p* < 0.0001; ORR 74% vs. 62%, respectively, *p* = 0.03) [28]. Results from a network meta-analysis by Ando et al. showed that alectinib and brigatinib as first-line are similar for PFS, both in all patients (HR: 1.171, 95% CI 0.702–1.841) and in the subgroup with CNS metastases (HR: 0.601, 95% CI 0.212–1.362) [29]. From the indirect comparison of these two ALK-i, in the subgroup with CNS metastases the difference results not statistically significant, although there is a trend in favor of a greater benefit with brigatinib. For this reason, both alectinib and brigatinib represent the therapeutic standard in ALK-rearranged NSCLC and the choice of one or of the other molecule is guided by the toxicity profile of each drug.

### 3.5. Ensartinib

Ensartinib has been studied in Exalt-3 trial, where it has been compared to crizotinib as first-line treatment [30]. Median PFS resulted significantly longer with ensartinib than with crizotinib (25.8 months vs. 12.7 months, HR: 0.51, 95% CI 0.35–0.72, *p* < 0.001) and intracranial disease was well controlled by ensartinib. Currently, ensartinib is approved in China (but not by FDA and EMA) for treatment in advanced ALK-rearranged NSCLC patients.

### 3.6. Lorlatinib

Lorlatinib, a third-generation’s ALK and ROS-1 inhibitor, as monotherapy is currently approved for the treatment of adult patients with ALK-positive advanced NSCLC, whose disease progressed on crizotinib and at least one other ALK inhibitor or on alectinib or ceritinib as first-line for metastatic disease [31]. Currently, lorlatinib has not yet been approved after failure of first-line therapy with brigatinib, but real-life studies are highlighting its activity also in this setting [32,33]. More recently, lorlatinib has been approved by FDA and EMA in first-line in NSCLC patients: the decision was based on findings from the phase 3 CROWN trial that compared lorlatinib to crizotinib in patients with ALK-translocated advanced NSCLC, not previously treated. Authors showed a 72% reduction in risk of disease progression or death in favor of lorlatinib compared to crizotinib. Moreover, lorlatinib resulted in an ORR of 76% compared to 58% of crizotinib (odds ratio: 2.25, 95% CI 1.35–3.89). In a cohort of patients with brain metastases at baseline, investigators reported an intracranial response rate of 82% (with complete response in 71% of cases) and 23% in the lorlatinib and crizotinib arms, respectively [34]. Lorlatinib showed an optimal penetration of the blood-brain barrier and can overcome multiple ALK resistance mutations, for which other ALK-i are ineffective.

### 3.7. ALK-i Safety and Toxicity

All ALK-i are manageable and safe and they are mainly characterized by hematological, gastrointestinal, ocular and hepatic adverse events (AEs). Several studies agree that discontinuations due to AEs remain at low incidence if ALK-i toxicities are correctly managed with dose modification and medical therapy.

Toxicity profiles slightly differ based on the specific drug. The most common AEs of alectinib are increase in liver enzymes, peripheral edema, myalgia and anemia. Conversely, lorlatinib is mainly characterized by hyperlipidemia, edema, peripheral neuropathy, increased weight and cognitive effects (including memory impairment, disturbance in attention, confusion, anxiety and depression). In the CROWN trial [35], AEs that were more common with crizotinib (than with lorlatinib) included diarrhea, nausea, vision disorder, vomiting, increase in liver enzymes and constipation. In the same study, a higher percentage of Grade 3 and Grade 4 AEs occurred in patients who received lorlatinib, compared to those receiving crizotinib (72% vs. 56%).

In a systematic review and network meta-analysis, comparing the effectiveness and safety of multiple first line treatment options, ceritinib showed the highest rate of Grade 3 AEs (60%), mainly gastrointestinal and liver toxicity, while alectinib resulted the safest one [36].

### 3.8. Treatment Sequence with ALK-i: A Challenge

The current scenario of therapeutic management of ALK-rearranged NSCLC patients is really challenging and the optimal sequence of ALK-i is currently an ongoing field of investigation. Given their efficacy in survival and intracranial activity, alectinib and brigatinib are currently preferable in first-line treatment, especially for patients with brain metastases: the choice of first-line ALK-i for each patient has to be guided by CNS involvement and toxicity profile [29].

A recent meta-analysis and systematic review conducted by Giunta et al. [37] reported a clear advantage for next-generation ALK-i compared to crizotinib and chemotherapy, with regard to PFS, ORR, DCR and intracranial responses. Moreover, OS seems to be improved by upfront treatment with next-generation ALK-i but the authors claim that data for all examined trials are still immature.

Actually, a second-generation TKI as upfront treatment is preferred to sequential treatment with first-generation followed by second-generation ALK-i. For patients that have received crizotinib as first-line treatment, second-generation TKIs maintain its efficacy in subsequent lines. Moreover, the choice between using a third generation TKI in the first line or after progression to a second generation TKI represents a current question for clinicians [37], in order to delay the use of chemotherapy and to guarantee patients the best overall survival.

## 4. Prognostic and Predictive Factors in Patients Treated with ALK-i

Several studies highlighted the potential role of clinical and laboratory factors in influencing prognosis of NSCLC patients treated with ALK-i. All trials that first proved crizotinib [23], ceritinib [25], alectinib [27], brigatinib [38], lorlatinib [35] and ensartinib [30] activity in ALK-rearranged NSCLC confirmed their efficacy in all investigated subgroups irrespective of age, sex, smoking habit or ethnicity. However, among these trials only ALEX and eXalt3 enrolled a high number of Asian subjects, so results of all trials involving ALK-i may not be fully applicable to Asian populations.

### 4.1. Role of Ethnicity

The ASCEND-4 trial, which first proved the superiority of ceritinib over standard chemotherapy in ALK-rearranged NSCLC, showed a greater benefit for ceritinib both in Caucasian and Asian populations. In these two subgroups, efficacy seems to be lower in Asian patients (HR: 0.66, 95% CI 0.41–1.06), with a PFS of 26.3 months, compared to 16.4 months in Caucasians (HR: 0.44, 95% CI 0.30–0.66) that made up most of the population considered. Similarly, the CROWN trial demonstrated that lorlatinib provides a lower rate of disease progression in non-Asian subjects (HR: 0.19, 95% CI 0.11–0.32) than in Asian subjects (HR: 0.47, 95% CI 0.27–0.82). However, at least for alectinib and brigatinib, J-ALEX [39], ALESIA [40] and J-ALTA [41] further validated their use in previously treated Asian patients, while ensartinib even performed better in Asian than in non-Asian subjects (HR: 0.32, 95% CI 0.19–0.55 vs. HR: 0.61, 95% CI 0.34–1.11) in eXalt3 study. To this regard, an interesting meta-analysis by Kuan Li Wu et al. [42] considering Asian patients from six different studies (CROWN, ALTA1L, ALEX, J-ALEX, ALESIA and eXalt3), recently confirmed that, although all ALK-i included in the analysis proved superior to crizotinib in treatment naïve NSCLC patients, ensartinib may be the best choice in first line in this population for PFS. Alectinib was second to ensartinib in terms of PFS, but it demonstrated the best ORR in a greater number of patients analysed. Moreover, low-dose alectinib (300 mg twice daily) was also found to be quite effective (specifically, in Japanese patients). Albeit this study has some limitations due to the unbalance between Asian patients treated with the different TKIs (a fact that favors alectinib’s greater numbers and may have over-estimated its efficacy in this population), it still represents a useful addition to our knowledge of these drugs activity in ALK-translocated NSCLC patients.

### 4.2. Inflammatory and Nutritional Laboratory Markers

Inflammation is deeply related with tumor development and progression, and the importance of immunological and nutritional laboratory markers as well as prognostic scores, considering serum albumin, neutrophil-to-lymphocyte ratio (NLR), platelet-to-lymphocyte ratio (PLR), systemic immune inflammation index (SII), has already been investigated in different tumours and also in NSCLC treated with immunotherapy [43,44] or target therapies. In a large cohort of patients with ALK-rearranged NSCLC treated with first line crizotinib, pre-treatment hypoalbuminemia was correlated with a shorter PFS [45]. Additionally, elevated NLR, PLR and decreased haemoglobin have been demonstrated to negatively affect PFS but also OS in retrospective analyses [46], though other experiences only confirmed PLR to affect prognosis of NSCLC patients in the same setting [47]. Another scoring system, the Pan-Immune-Inflammation Value (PIV) takes into account white blood-cell count and platelets (calculated as multiplication of neutrophil count, platelet count, monocyte count, divided by lymphocyte count) and has already been validated in different solid tumours such as metastatic melanoma and breast cancer [48,49]: it has showed promising prognostic power also in NSCLC mainly treated with crizotinib and alectinib in a retrospective analysis by Xinru Chen et al. [50]. Focusing on alectinib, Takeda et al. [51] recently evaluated pre-treatment and post-three weeks therapy values of PLR, systemic immune-inflammation index (SII) (calculated as multiplication of neutrophil count and platelets count, divided by lymphocyte count), and prognostic nutrition index (PNI, which considers the serum albumin concentration and peripheral blood lymphocyte count). Baseline PLR, SII, PNI and the 3-week values for SII and PNI were significantly associated with longer PFS, thus providing a solid proof of the importance of the baseline nutritional and inflammatory status during alectinib treatment.

However, the role of inflammation indexes in ALK-rearranged patients still has to be validated in larger cohorts and, in particular, in innovative drugs such as brigatinib or lorlatinib that have only been recently introduced in clinical practice.

## 5. Mechanisms of Resistance to ALK-i

The benefits of all ALK-i are limited by the onset of mechanisms of drug resistance that inevitably occurs after a variable period of treatment. Resistance to ALK-i treatment can be distinguished in primary and acquired resistance: primary resistance occurs when a patient fails to respond to target treatment from the beginning of ALK-i therapy and disease progression to ALK-i occurs within 3 months. Primary resistance implies a de novo ALK resistance mutation and seems to be rare in ALK-rearranged NSCLC (<3–5% of cases) [52].

**Figure 3:**
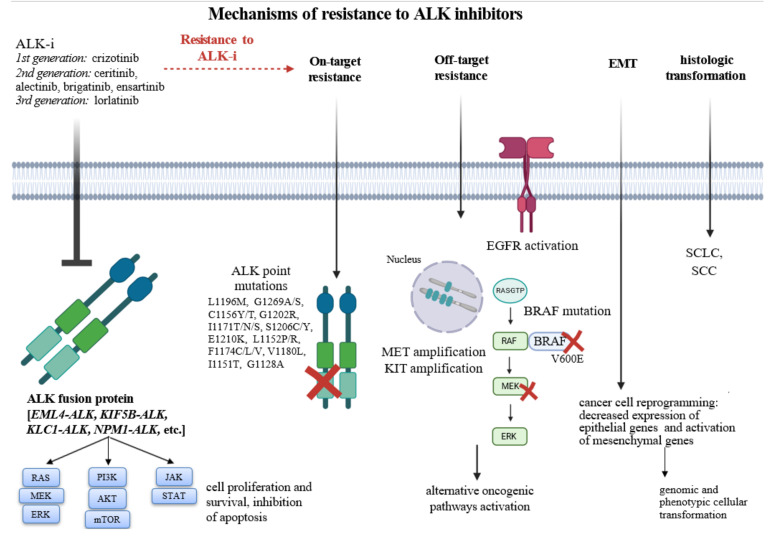
Mechanisms of resistance to ALK- inhibitors, including on-target and off-target molecular mechanisms. Abbreviations: ALK-i, ALK inhibitors; EMT, epithelial-to-mesenchymal transition; SCLC, small-cell lung cancer; SCC, squamous cell carcinoma.

The mechanism of acquired resistance to ALK-i can be divided into two categories, including ALK-dependent alterations (on target) and ALK-independent alterations (off target), such as bypass signaling or epithelial-to-mesenchymal transition (Figure 3). Specific genome alterations in *ALK* or other genes are affected by the specific ALK-i and the treatment sequence.

### 5.1. On-Target Resistance Mechanisms

On-target resistance mechanisms lead to re-activation of ALK kinase activity and over-stimulation of downstream signalling. Resistance mutations can interfere with ALK-i or ATP binding to kinase or can induce structural changes on the ALK kinase domain [53,54]. Resistance mechanisms to ALK-i imply ALK mutations in variable proportion of patients based on specific ALK-i, involving nearly 30%, 50% and 70% of patients treated with crizotinib, second generation ALK-i and lorlatinib, respectively. [55,56] The most common ALK mutations found in patients treated with ALK-i are: L1196M, G1269A/S, C1156Y/T, 19 G1202R, I1171T/N/S, S1206C/Y, E1210K, L1152P/R, F1174C/L/V, V1180L, I1151T and G1128A [54,57].

In particular, G1202R mutation is found in 2% of the samples of patients progressed to crizotinib, while it is the most common resistance ALK mutation after second-generation ALK-i treatment, occurring in 21%, 29% and 43% of patients treated with ceritinib, alectinib and brigatinib, respectively [58]. Interestingly, lorlatinib is the only agent effective against G1202R mutation: it has been described by Zhou et al. that the presence of G1202R mutation reduces lorlatinib binding affinity to ALK with a mechanism of steric hindrance but, despite that, lorlatinib maintains its potent activity to achieve clinical benefit [59,60]. Lorlatinib demonstrated strong activity also for common resistant mutations such as L1196M and G1269A, but it is yet to be proven if the characteristic of overcoming these mutations reflects in better clinical benefit for NSCLC patients, compared to other ALK-i.

After treatment with lorlatinib or sequential treatment with ALK-i, compound ALK mutations have been detected in patients that developed resistance, adding complexity to the spectrum of detectable cancer cell subpopulations. Recondo et al. identified a combination of 1196M/D1203N, F1174L/G1202R, and C1156Y/G1269A mutations and Zhou et al. described a case of G1202R/S1206Y double mutation, confirming the stepwise accumulation in lorlatinib-resistance patients [59,61]. In fact, when patients receive sequential ALK-i treatment, cancer cells accumulate more than one resistance mutation, increasing intercellular genomic heterogeneity. Gainor et al. identified a case with compound mutation in a patient treated with crizotinib and subsequently brigatinib: a re-biopsy performed within the same anatomic site demonstrated acquired E1210K and S1206C resistance mutations, revealing an evolutionary picture in the same patients [58].

As well as point mutations, ALK-A has also been described by Katayama et al. as a possible resistance mechanism to crizotinib, even if with low incidence [56].

### 5.2. Off-Target Resistance Mechanisms

Off-target mechanisms were shown to be responsible for resistance to ALK-i in up to 55% of both tumor and blood samples, in a large cohort of ALK-rearranged NSCLC patients [55]. Other authors reported an incidence of 60–70% and 25–45% of ALK independent resistance in patients treated with crizotinib or second-generation ALK-i, respectively [53]. The activation of the bypass signaling pathways is the main ALK-independent resistance mechanism to ALK-i, including EGFR signaling [62], amplification of KIT, MET amplification [63] and BRAF V600E mutation [64]. The incidence of bypass signaling activating mutations has been reported in small cohorts of patients and varies according to the drug and sample used (tissue or blood) and is more common in patients treated with sequential ALK-i than in patients receiving crizotinib alone [65].

Hystological changes belong to off-target resistance mechanisms and involve morphological transformation of cancer cells in histotypes different from the original one. ALK-i resistance may be due to the conversion to small cell lung cancer (SCLC) or squamous cell carcinoma (SCC): Kaiho et al. reported a rare case of a patient with diagnosis of ALK-rearranged adenocarcinoma that transformed to SCC after alectinib and was not responsive to ceritinib. [66] Another case described a patient that developed both G1202R mutation and transformation in SCLC after sequential treatment with ceritinib and lorlatinib. In this case, authors showed that deletion of p53 and retinoblastoma (RB) genes is important for SCLC transformation, although the transformation mechanism is not fully understood [67].

Epithelial-to-mesenchymal transition (EMT) consists in a genomic and phenotypic transformation of epithelial cells that lose their polarity and intercellular connections, acquiring migratory and invasive ability. EMT is based on a genomic reprogramming of cancer cells, which was associated with a gain of expression of mesenchymal markers and decreased expression of epithelial markers, such as E-cadherin. Four molecular pathways have been associated with EMT and with ALK-i resistance, including proteoglycan in cancer, HIF-1 signaling, FoxO signaling, and extracellular matrix receptor interactions [68].

## 6. Current and Future Applications of Liquid Biopsy in ALK-Rearranged NSCLC

In recent years, liquid biopsy progressively acquired a greater role in solid cancer, especially in colorectal cancer and NSCLC. It consists in sampling and analysis of analytes and small molecules from different biological fluids, mostly blood but also urine or cerebrospinal liquid [69,70]. From these easy-to-access samples, several kinds of molecules can be identified, among the most interesting for oncological purposes circulating tumor cells (CTCs) and circulating cell-free DNA (cfDNA), which in cancer patients includes circulating tumour DNA (ctDNA) [71]. ctDNA is thought to reflect the tumor heterogeneity or dinamics, as well as presence of tumor-specific subclonal DNA mutation [72,73,74].

### 6.1. Cancer Detection and Characterization

Liquid biopsy represents a non-invasive procedure, potentially applicable to cancer detection and characterization [75]: in the field of NSCLC management, it is recommended by international guidelines [76] as molecular testing complementary to (but not replacement of) tissue-based diagnostics. The application of liquid biopsy detains a role in case of inadequate tumor-tissue sampling or when tissue biopsy is unfeasible because of a patient’s poor Performance Status or unachievable tumor site [77].

The advantages of liquid biopsy, compared to tissue sample, are represented by lower costs and no risk of major complications for the patient. The total amount of ct-DNA and CTCs may be representative of multiple tumor sites within the same patient and give a comprehensive view of the entire tumor genome [77]. However, the search for tumoral nucleic acids in blood specimens may reveal DNA from non-tumor sources [78], also due to technique difficulty or improper blood sample processing. Low-burden or slow-growing cancers may release only undetectable numbers of tumor DNA into the circulation [73], with a risk of more false negative respect to tissue biopsy [79,80,81]. Finally, only tissue samples can detect a transformation of tumor cells into small/squamous cell carcinoma or the presence of epithelial-mesenchymal transformation.

### 6.2. Monitoring Response to Treatment

In the ALK-rearranged NSCLC setting, liquid biopsy, mainly through ctDNA [73], progressively gained a routine application in the monitoring response to treatment and in early detection of tumor remission and molecular resistance to ALK-i. This method provides a gene profiling of oncogene-addicted NSCLC patients who progressed to first-line target treatment, thus identifying acquired ALK resistance mutations and other gene alterations responsible for therapy failure [82,83].

Looking at comparable clinical outcomes of ALEX and BFAST studies, which allocated ALK-mutated NSCLC patients to alectinib treatment based on tissue or liquid biopsy results, respectively, it can be deduced that also ALK rearrangement in ctDNA can be considered a valid approach to select patients suitable for alectinib in first-line therapy [27,84,85]. Liquid biopsy has been confirmed as a valid method to identify patients to whom ALK-i should be administered.

In a phase II trial, involving 121 patients treated with lorlatinib, the dynamics of plasma ALK rearrangement showed a significant correlation with deeper response and longer PFS: so, ctDNA dynamics may predict lorlatinib efficacy [86]. Moreover, the same authors demonstrated that, in NSCLC patients failed to one or more second-generation ALK-i, ALK mutation status revealed by liquid biopsy is predictive of response to treatment with lorlatinib (ORR 62% vs. 32%, for ALK-positive mutation and ALK negative patients, respectively), similar to tissue genotyping (69% vs. 27%) [87].

Another group [88] demonstrated that ct-DNA detection at baseline occurred in 51% of plasma samples (from ALK-rearranged NSCLC patients) and correlated with shorter PFS, compared to patients without detectable mutations at baseline. The presence of ALK resistance alterations, suggestive of early molecular progression, was revealed in 19 out of the 43 patients (44%).

Dagogo et al. demonstrated that plasma genotyping detects more ALK mutations than tumor genotyping. In particular, a higher proportion of alectinib or lorlatinib-resistant patients had ≥2 ALK mutations in plasma samples, with respect to the one emerged by tissue sample. So, liquid biopsy can provide a more detailed frame of intratumoral heterogeneity and detect genomic alterations across multiple disease sites [89].

In the setting of ALK-i resistance, liquid biopsy could direct the choice of subsequent treatments, due to the detection of potentially actionable molecular alterations in advanced NSCLC [58,90].

Sanchez-Herrero et al. [91] analyzed ct-DNA profiling from 24 advanced ALK-rearranged NSCLC patients after the failure of ALK-i treatment: at least one ALK mutation was detected in 38.5% of plasma samples and the G1202R mutation emerged in four patients who progressed on alectinib or ceritinib. Somatic mutations were identified in 14 genes: a deletion in exon 19 of the EGFR gene, a non-V600 BRAF mutation, and the F129L mutation in MAP2K1 (MEK1) were shown in four patients who do not benefit from ALK-i, while molecular alterations were detected different pathways involving *TP53, ALK, PIK3CA, SMAD4, FGFR2, FGFR3, IDH2, MYC, MET, CCND3* and *CCND1* genes. These findings reveal a new scenario that comprises potential ALK-i resistance mutations in ALK-not related signaling and that may explain primary or secondary resistance to target therapy.

In a large phase 2 study [92], involving 168 ALK-rearranged NSCLC patients who progressed on crizotinib and were treated with ensartinib, ct-DNA was analyzed at baseline and a progression disease with a 212-gene panel. At the time of the progression of the disease, 41.7% of the patients had secondary ALK mutations: the most common ones were G1269A, G1202R and E1210K, whose frequency increased markedly compared with baseline. Moreover, baseline TP53 mutations (20.2%) significantly correlated with inferior PFS and higher mutation load: authors also affirmed that patients with TP53 mutations had significantly higher incidence of resistant secondary ALK mutations than those without TP53 mutations.

This evidence points out that liquid biopsy represents a simple and non-invasive tool to monitor ALK-rearranged disease response during treatment, to identify early clinical progression and mechanisms of resistance to ALK-i [81]. Ct-DNA analysis accurately reflects tumor heterogeneity and, by tracking serially genetic mutations from blood samples, allows prompt therapeutic decisions, tailored to the genetic profile of the disease progression [93]. NGS of ct-DNA for detection of ALK fusions or other alterations represents an emerging alternative to tissue-based methodologies such as fluorescence in situ hybridization (FISH), immunohistochemistry (IHC), and tissue-based NGS [94] that could support clinicians in daily clinical practice to tailor subsequent treatment strategies on molecular tumor characteristics.

## 7. Combination Treatment Strategies and Future Directions

In order to further improve survival and clinical benefit in patients harboring ALK rearrangements, different therapeutic strategies of sequential or combined ALK-i or combination treatment of ALK-i with chemotherapy, immunotherapy or other target agents are being tested in ongoing trials (Table 1).

Nowadays, there is a great deal of attention being paid to the development of new drug combinations to overcome the different resistance mechanisms occurring after ALK-i treatment. High hopes are placed in fourth generation ALK TKIs that seem capable of targeting acquired compound ALK mutations as well as a wide range of single ALK mutations. A phase I/II clinical trial of ALK-i TPX-0131 for previously treated ALK-rearranged NSCLC patients is currently ongoing (NCT04849273) [105], while another drug (NVL-655) has demonstrated activity in lorlatinib-resistant models of NSCLC. Moreover, patients experiencing disease progression to alectinib due to MET amplification may be sensitive to the combination of alectinib and crizotinib [107] though available evidence is limited.

Following evidence supporting the use of ramucirumab in combination with erlotinib in EGFR mutated NSCLC [108], angiogenesis inhibitors have also been evaluated in ALK-rearranged NSCLC. In particular, a single Chinese observational study [109] demonstrated the potential effectiveness of bevacizumab, an antibody directed against vascular endothelial growth factor (VEGF), in combination with crizotinib, providing a PFS of 14 months in 12 patients enrolled with a manageable safety profile. Only two patients interrupted treatment due to liver toxicity and haemoptysis. Consequently, trials that combined ALK-i with other cytotoxic agents, such as chemotherapy, are ongoing. NCT04227028 [95] and NCT02521051 [96] are studying bevacizumab in combination with second generation TKIs brigatinib and alectinib, respectively.

Immunotherapy has radically changed the treatment paradigm in different solid tumors but immune-checkpoint inhibitors (ICIs) activity in NSCLC expressing oncogenic driver mutations is limited. ICI monotherapy after progression to ALK inhibition demonstrated poor outcomes, both in terms of PFS and ORR in retrospective experiences [110,111]. Nevertheless, interest in the potential application of immunotherapy in this setting kept growing and led to trials investigating different combinations with ALK-i. In fact, though an interesting activity of ICI-TKI doublet, in particular considering nivolumab plus crizotinib [112] and ceritinib [113], was proved with an ORR from 40% to 80% depending on TKIs dose level, high-grade toxicity led to frequent treatment discontinuations or dosing remodulation. In this regard, the study from Patel et al. [114] enrolled patients with treatment naïve ALK-rearranged stage IV NSCLC treated with crizotinib plus pembrolizumab doublet and has been terminated early due to elevated alanine aminotransferase level (25%), elevated gamma-glutamyl transferase level (22%), elevated amylase level (14%), elevated lipase level (11%), and maculopapular rash (11%). Similarly, a phase I trial of erlotinib or crizotinib in combination with the CTLA-4 inhibitor ipilimumab [115] was interrupted. Unexpectedly, NCT02013219 [97] evaluating atezolizumab with alectinib (alectinib 600 mg bid for 7 days for safety evaluation, followed by alectinib 600 mg bid with atezolizumab 1200 mg q3w) has instead been completed with an acceptable safety and a 57% grade 3 event rate. At a median follow up of 29 months, ORR was 86% (95% CI 64–97), median progression-free survival was not estimable (95% CI 13 months-not estimable) and median OS resulted not evaluable (95% CI 33 months-not estimable). All 21 enrolled patients had discontinued study treatments.

Focusing on chemotherapy, it is already known that pemetrexed is quite effective in ALK-rearranged tumors. In 2013, the control arm of PROFILE 1007 trial that first demonstrated crizotinib activity in NSCLC showed a median PFS of 4.2 months in the pemetrexed arm and 2.6 months in the docetaxel arm. It can be deduced that pemetrexed was more effective than docetaxel. Consequently, Camidge et al. [116] examined data from 89 advanced NSCLC patients; median PFS was 9 months (95% CI 3–12) in the ALK positive, 5.5 months (95% CI 1–9) in the EGFR mutant, 7 months (95% CI 1.5–10) in the KRAS mutant, and 4 months (95% CI 3–5) in the wild type patients. After multivariate analysis, ALK was the only driver mutation associated with prolonged PFS in the pemetrexed chemotherapy regimen. Another study from Lee et al. [117] found a better ORR (46.7% in ALK-positive, 16.2% in the EGFR mutant, and 4.7% in the KRAS mutant patients) and time to progression (9.2 months in the ALK-positive, 1.4 months in the EGFR mutant, and 2.9 months in the KRAS mutant patients) in ALK-rearranged NSCLC treated with pemetrexed, regardless of treatment line. In order to explore the potential of standard chemotherapy with platinum and pemetrexed combined with ALK-i brigatinib, the Japanese jRCTs041210103 trial [118] is actually ongoing. In addition, NCT04837716 trial [101] is evaluating another ALK-i, ensartinib, combined with bevacizumab and four cycles of standard chemotherapy with carboplatin and pemetrexed in order to determine safety and identify the recommended dose of ensartinib. Three ongoing clinical trials in second-line settings after ALK-i progression will, instead, determine the efficacy and tolerability of chemotherapy with platinum and pemetrexed in combination with atezolizumab +/− bevacizumab (NCT04042558) [119], atezolizumab + bevacizumab + carboplatin and paclitaxel (NCT03991403) [120] and pembrolizumab + bevacizumab + standard chemotherapy (NCT05266846) [121].

Finally, Voena et al. [122] described the efficacy of an ALK vaccine in combination with ALK-i treatment in mouse model. ALK vaccine is thought to induce a strong systemic and intratumoral immune response, eliciting an ALK-specific cytotoxic response. The combination with ALK-i can significantly modulate the tumor immune microenvironment and induce long-lasting immune response against cancer cells. The Authors also supposed a possible enhancement of antitumoral activity by adding an anti-PD-1/PD-L1 or anti-CTLA4 drug to ALK vaccine and ALK-i.

## 8. Conclusions

The clinical outcome for ALK-rearranged NSCLC patients has dramatically changed in recent years thanks to the introduction in clinical practice of efficient ALK-i, which is able to significantly stop tumor growth and to guarantee patients long survival with high quality of life. Since then, the current challenge is represented by the emergence of resistance mechanisms capable of evading ALK-i activity, and many questions (not completely resolved) have emerged regarding the best upfront strategy and therapeutic sequence. Indeed, second and third-generation ALK-i as first line confirmed a greater clinical benefit when compared to crizotinib and chemotherapy, but head-to-head comparison studies between next-generation ALK-i are still lacking. In this setting, growing evidence shows liquid biopsy as a reliable tool to guide the choice of personalized treatments and to point out underlying molecular mechanisms of clinical progression disease.

Finally, the development of new drugs and the combination between ALK-i and other target agents or ICI most likely will represent the way to move forward in this exciting field of NSCLC research.

## Figures and Tables

**Table 1:** Ongoing clinical trials, including ALK-i in combination with angiogenesis inhibitors, ICIs, chemotherapy or MEK inhibitors and novel oral ALK-i.

**Clinical Trials with Angiogenesis Inhibitors and ALK-i**							
**Trial Name**	**Intervention**	**Phase**	**Design**	**Center**	**Planned Participant Enrollment**	**Status**	**Primary** **Objective**
NCT04227028 [95]	Brigatinib + Bevacizumab	I	Single group assignment, open label	Multicenter, 4 sites	31	Recruiting	Recommended dose, safety
NCT02521051 [96]	Alectinib + Bevacizumab	I/II	Single group assignment, open label	Multicenter, 2 sites	43	Recruiting	Recommended dose, safety
**Clinical Trials with ICIs and ALK-i**							
**Trial Name**	**Intervention**	**Phase**	**Design**	**Center**	**Planned Participant Enrollment**	**Status**	**Primary Objective**
NCT02013219 [97]	Atezolizumab + Erlotinib or Alectinib	I	Non-randomized, sequential assignment, open label	Multicenter, 17 sites	52	Completed	Safety, recommended dose, pharmacokinetics
NCT02393625 [98]	Nivolumab + Ceritinib	I	Non-randomized, parallel assignment, open label	Multicenter, 11 sites	57	Active, not recruiting	MTD and/or recommended dose for expansion, overall response rate
NCT02584634 [99]	Avelumab + Erlotinib or Lorlatinib	I/II	Non-randomized, open label	Multicenter, 21 sites	43	Active, not recruiting	DLTs, ORR, CR
**Clinical Trials with Chemotherapy and ALK-i**							
**Trial Name**	**Intervention**	**Phase**	**Design**	**Center**	**Planned Participant Enrollment**	**Status**	**Primary Objective**
NCT05200481 [100]	Carboplatin + Pemetrexed + Brigatinib	II	Randomized, open label, non-comparative	Multicenter, 30 sites	110	Recruiting	PFS, OS, ORR
NCT04837716 [101]	Carboplatin + Pemetrexed + Bevacizumab + Ensartinib	I	Single group assignment, open label	Single center	12	Recruiting	Safety, recommended dose
**Clinical Trials with MEK Inhibitors and ALK-i**							
**Trial Name**	**Intervention**	**Phase**	**Design**	**Center**	**Planned Participant Enrollment**	**Status**	**Primary Objective**
NCT03202940 [102]	Alectinib + Cobimetinib	I/II	Single group assignment, open label	Single center	31	Recruiting	MTD
NCT04005144 [103]	Brigatinib + Binimetinib	I	Single group assignment, open label	Single center	18	Recruiting	Safety, tolerability
**Clinical Trials with ALK-i Combination**							
**Trial Name**	**Intervention**	**Phase**	**Design**	**Center**	**Planned Participant Enrollment**	**Status**	**Primary Objective**
NCT04292119 [104]	Lorlatinib + Crizotinib or Binimetinib, or TNO155	Ib/II	Non-randomized, parallel assignment, open label	Multicenter, 2 sites	96	Recruiting	MTD, ORR
**Clinical Trials with Novel Oral ALK-i**							
**Trial Name**	**Intervention**	**Phase**	**Design**	**Center**	**Planned Participant Enrollment**	**Status**	**Primary Objective**
NCT04849273 [105]	TPX-0131	I/II	Single group assignment, open label	Multicenter, 15 sites	210	Recruiting	Safety, recommended dose, overall response rate
NCT05384626 [106]	NVL-655	I/II	Non-randomized, open label, sequential assignment	Multicenter, 6 sites	214	Recruiting	DLTs, RP2D, ORR

Abbreviations: ALK-i, ALK inhibitors; CR, complete response; DLTs, dose-limiting toxicities; ICIs, immune checkpoint inhibitors; MTD, maximum tolerated dose; ORR, objective response rate; OS, overall survival; PFS, progression free survival; RP2D, recommended phase 2 dose.

## Data Availability

The data presented in this study are available on request from the corresponding author.
